# Supplementary material for: Spicy Bitumen: Curcumin Effects on the Rheological and Adhesion Properties of Asphalt
Source: Materials (Basel). 2021 Mar 26;14(7):1622. doi: 10.3390/ma14071622 (PMC8036851; doi:10.3390/ma14071622)
Supplement: Supplementary file 1 [file materials-14-01622-s001.pdf]

materials

Suplementary Information

Spicy Bitumen: Curcumin effects on the rheological and adhesion properties of Asphalt

Abraham A. Abe [1], Cesare Oliviero Rossi [1,*], Paolino Caputo [1,*], Maria Penelope De Santo [2], Nicolas Godbert [3] and Iolinda Aiello [3,4]

[1] Dipartimento di Chimica e Tecnologie Chimiche, Università della Calabria, 87036, Rende, Italy; abraham.abe@unical.it
[2] Dipartimento di Fisica e CNR-Nanotec, Università della Calabria, via Bucci 31C, 87036, Rende, Italy; maria.desanto@fis.unical.it
[3] MAT-InLAB, LASCAMM CR-INSTM, Unità INSTM della Calabria, Dipartimento di Chimica e Tecnologie Chimiche, Università della Calabria, Ponte Pietro Bucci Cubo 14C, 87036, Rende, Italy; nicolas.godbert@unical.it (N.G.); iolinda.aiello@unical.it (I.A.)
[4] CNR NANOTEC-Istituto di Nanotecnologia U.O.S. Cosenza, 87036, Rende, Italy
* Correspondence: cesare.oliviero@unical.it (C.O.R.); paolino.caputo@unical.it (P.C.)

Figure S1. SEM and EDX composition of the residue obtained after calcination of turmeric powder.
Figure S2. FGC 2H bee-structure domains: topography (left), phase image (right)
Figure S3. AFM-3D representation of FGC 2H domains
Figure S4. AFM-Pure LC50
Figure S5. LC50 + 1% RGC
Figure S5. AFM-LC50 + 1% RGC
Figure S6. Boiling test LC50 and modified Bitumen.
Figure S7. Boiling test C170 and modified bitumen

Citation: Abe, A.A.; Oliviero Rossi, C.; Caputo, P.; De Santo, M.; Godbert, N.; Aiello, I. Spicy Bitumen: Curcumin Effects on the rheological and Adhesion Properties of Asphalt. *Materials* **2021**, *14*, x. https://doi.org/10.3390/ma14071622

Academic Editors: Valentina Grumezescu and Oana Gherasim

Received: 25 February 2021
Accepted: 20 March 2021
Published: date

Publisher's Note: MDPI stays neutral with regard to jurisdictional claims in published maps and institutional affiliations.

Copyright: © 2021 by the authors. Submitted for possible open access publication under the terms and conditions of the Creative Commons Attribution (CC BY) license (http://creativecommons.org/licenses/by/4.0/).

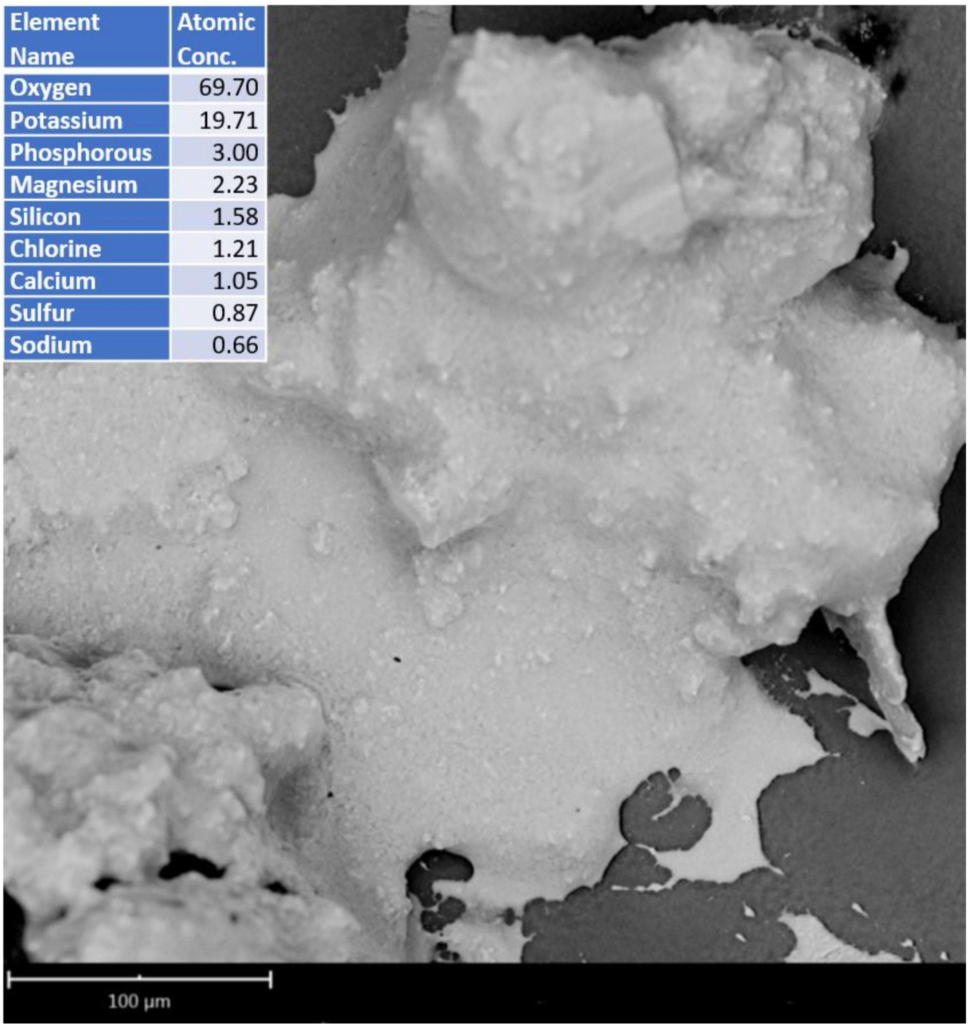

Element Name	Atomic Conc.
Oxygen	69.70
Potassium	19.71
Phosphorous	3.00
Magnesium	2.23
Silicon	1.58
Chlorine	1.21
Calcium	1.05
Sulfur	0.87
Sodium	0.66

Figure S1. SEM and EDX composition of the residue obtained after calcination of turmeric powder.

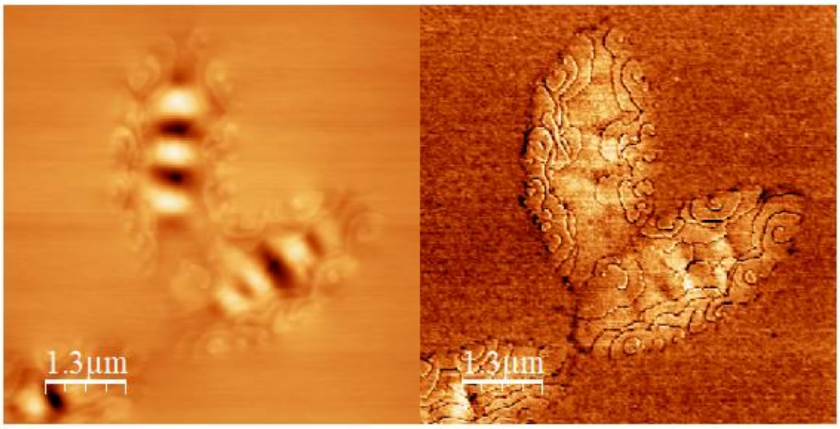

(left)	(right)

Figure S2. FGC 2H bee-structure domains: topography (**left**), phase image (**right**).

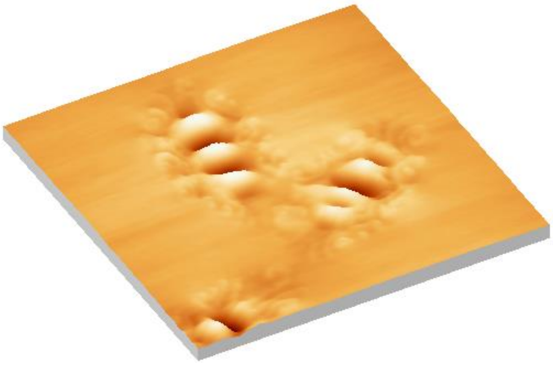

Figure S3. AFM-3D representation of FGC 2H domains.

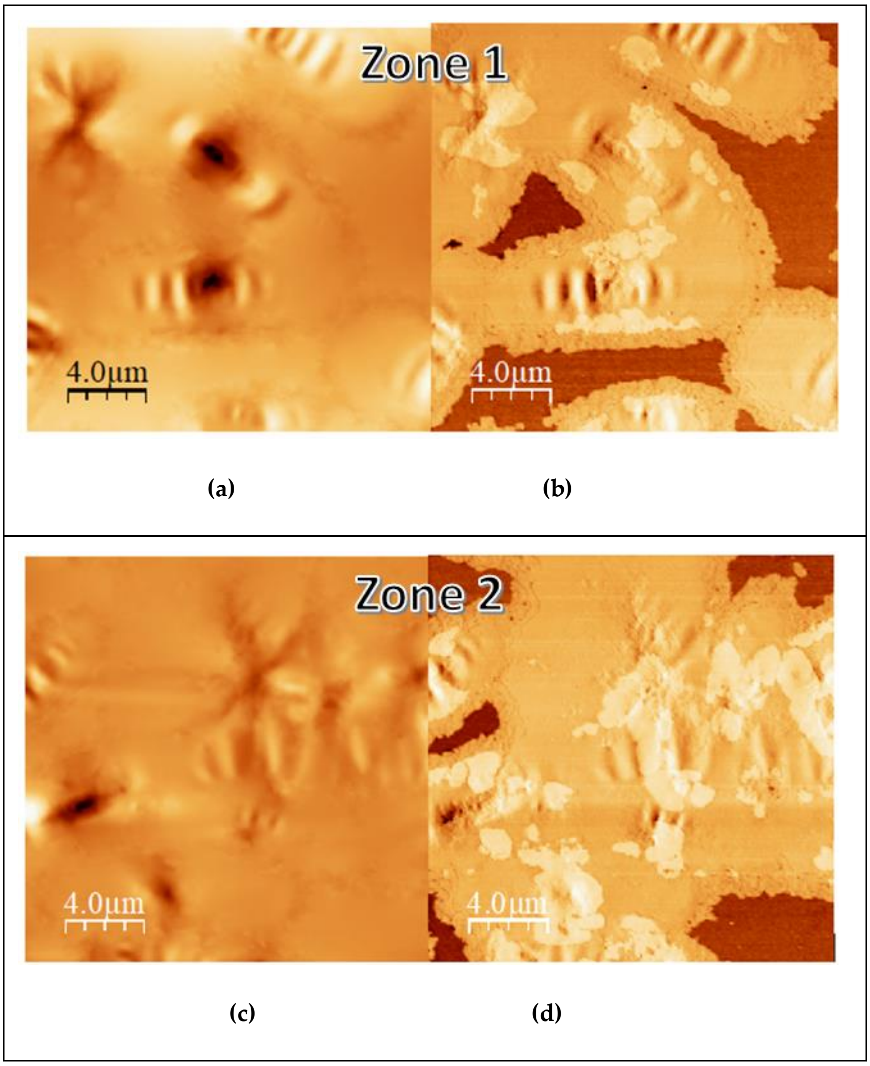

Figure S4. AFM-Pure LC50 sample surface. **(a,c)**: topography and **(b,d)**: phase image.

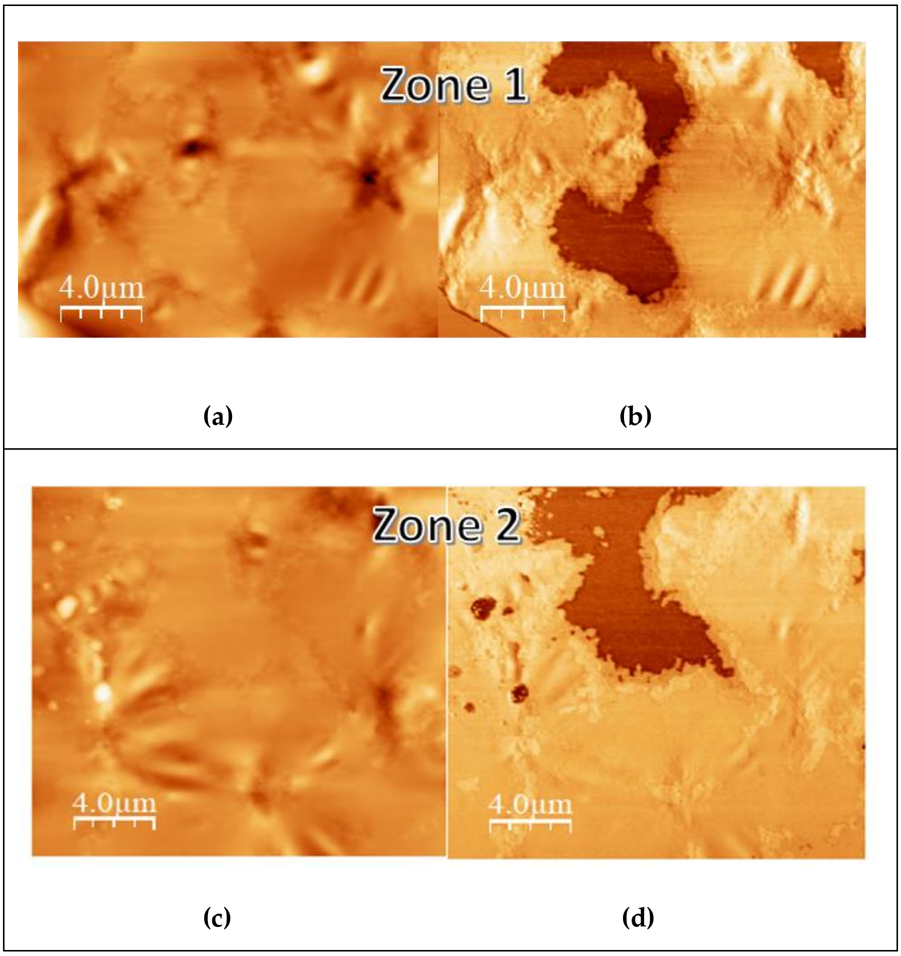

Figure S5. AFM-LC50 + 1% RGC sample surface. **(a,c)**: topography and **(b,d)**: phase image.

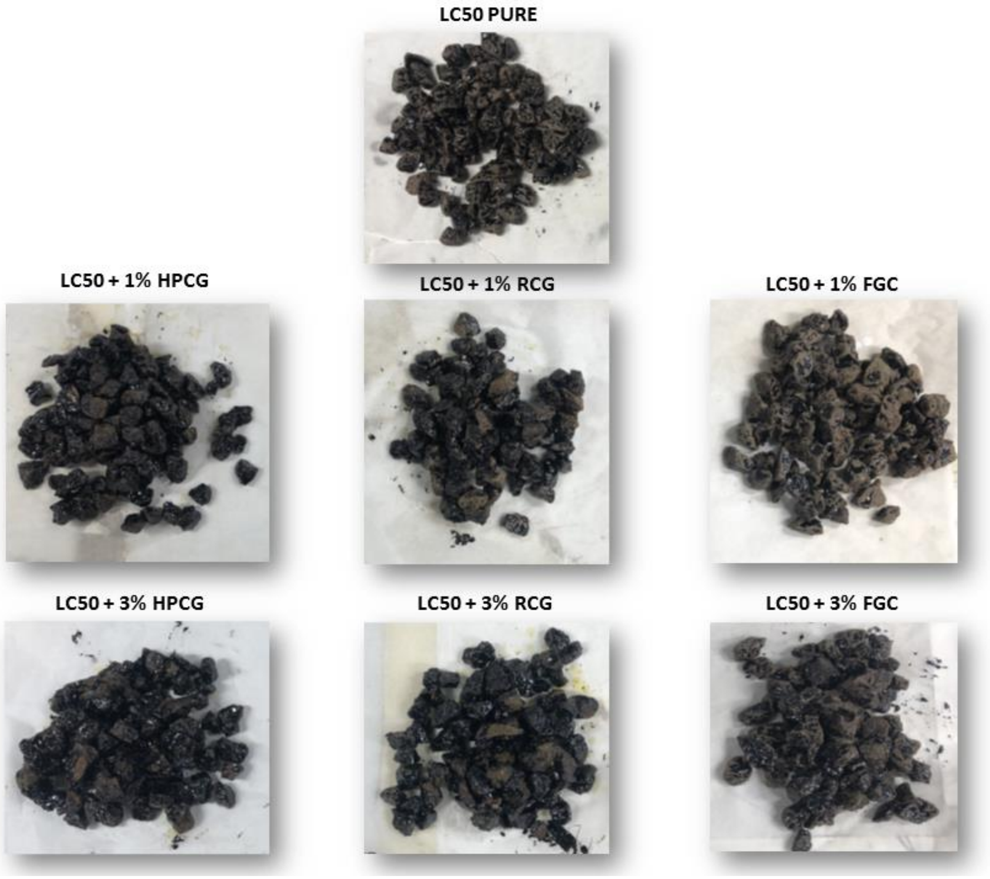

Figure S6. Boiling test LC50 and modified Bitumen.

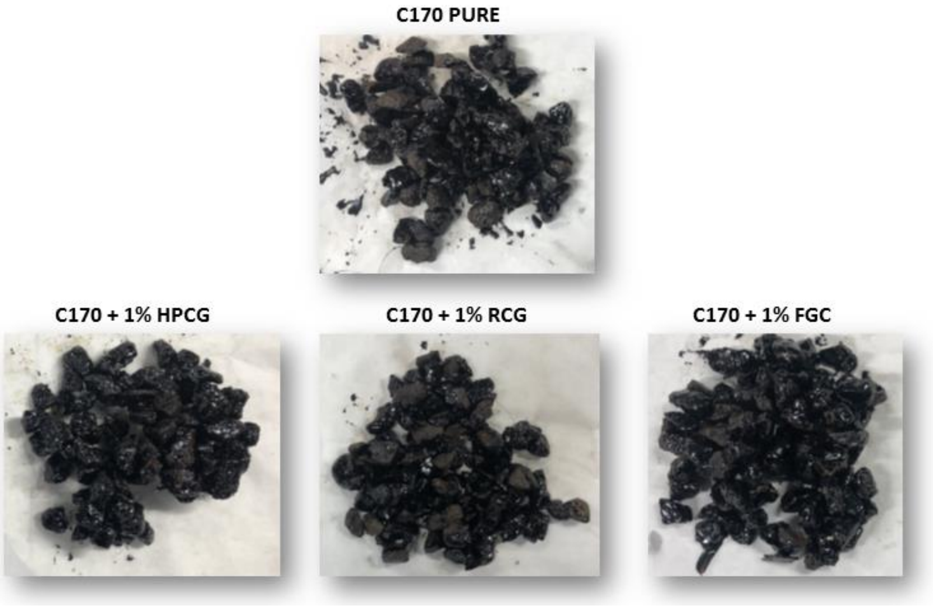

Figure S7. Boiling test C170 and modified bitumen.
